# Genetic association of *ERAP1* and *ERAP2* with eclampsia and preeclampsia in northeastern Brazilian women

**DOI:** 10.1038/s41598-021-86240-z

**Published:** 2021-03-24

**Authors:** Leonardo Capistrano Ferreira, Carlos Eduardo Maia Gomes, Priya Duggal, Ingrid De Paula Holanda, Amanda Samara de Lima, Paulo Ricardo Porfírio do Nascimento, Selma Maria Bezerra Jeronimo

**Affiliations:** 1 Department of Biochemistry, Center of Biosciences, Federal University of Rio Grande do Norte, Av. Sen. Salgado Filho, S/N, Campus Universitário - Lagoa Nova, Natal, RN 59078-900 Brazil; 2 Institute of Tropical Medicine of Rio Grande do Norte, Federal University of Rio Grande do Norte, Natal, Brazil; 3 Department of Biophysics and Pharmacology, Federal University of Rio Grande do Norte, Natal, Brazil; 4 Bloomberg School of Public Health, Johns Hopkins University, Baltimore, USA; 5 Department of Biochemistry, Federal University of Rio Grande do Norte, Natal, Brazil; 6 Maternidade Escola Januario Cicco, Federal University of Rio Grande do Norte, Natal, Brazil; 7 Institute of Science and Technology of Tropical Diseases (INCT-DT), Salvador, Brazil

**Keywords:** Genetic association study, Genetics research, Hypertension

## Abstract

The clinical spectrum of hypertensive disorders of pregnancy (HDP) is determined by the interplay between environmental and genetic factors, most of which remains unknown. *ERAP1, ERAP2* and *LNPEP* genes code for multifunctional aminopeptidases involved with antigen processing and degradation of small peptides such as angiotensin II (Ang II), vasopressin and oxytocin. We aimed to test for associations between genetic variants in aminopeptidases and HDP. A total of 1282 pregnant women (normotensive controls, n = 693; preeclampsia, n = 342; chronic hypertension with superimposed preeclampsia, n = 61; eclampsia, n = 74; and HELLP syndrome, n = 112) were genotyped for variants in *LNPEP* (rs27300, rs38034, rs2303138), *ERAP1* (rs27044, rs30187) and *ERAP2* (rs2549796 rs2927609 rs11135484). We also evaluated the effect of *ERAP1* rs30187 on plasma Ang II levels in an additional cohort of 65 pregnant women. The genotype C/C, in *ERAP1* rs30187 variant (c.1583 T > C, p.Lys528Arg), was associated with increased risk of eclampsia (OR = 1.85, *p* = 0.019) whereas *ERAP2* haplotype rs2549796(C)–rs2927609(C)–rs11135484(G) was associated with preeclampsia (OR = 1.96, corrected *p*-value = 0.01). Ang II plasma levels did not differ across rs30187 genotypic groups (*p* = 0.895). In conclusion, *ERAP1* gene is associated with eclampsia whereas *ERAP2* is associated with preeclampsia, although the mechanism by which genetic variants in ERAPs influence the risk of preeclampsia and eclampsia remain to be elucidated.

## Introduction

Hypertensive disorders of pregnancy (HDP) account for 14% of all maternal deaths^1^ and contribute to increase the cardiovascular risk in both mothers^2^ and offspring^3^. As other complex diseases, HDP have a broad clinical spectrum ranging from mild hypertension without proteinuria to severe proteinuria, and eventual seizures (*i.e.* eclampsia), or with hemolysis elevated liver enzymes and low platelet liver disease and severe inflammation (*i.e.* HELLP syndrome). Risk factors for preeclampsia, such as pre-gestational body mass index, nulliparity, change in partners, and advanced maternal age have been reported for different populations^4^. Regarding the genetics of preeclampsia, genes *INHBP*^5^, *FLT1*^6^ and *PLEKHG1*^7^ were identified from genome wide association studies, however, the genetic architecture underlying the disease mechanism remains largely unknown^8^.

Endoplasmic reticulum aminopeptidases -1 (ERAP1), -2 (ERAP2) and leucyl/cystinyl aminopeptidase (LNPEP), also known as A-LAP, L-RAP and P-LAP, respectively, are multifunctional enzymes belonging to the M1 family of aminopeptidases^9^. These aminopeptidases act in concert to trim peptides to be presented by the major histocompatibility complex (MHC) class I molecules^10^ and, in addition, they cleave a variety of bioactive peptides, including angiotensins, bradykinin, kallidin and oxytocin^11^. Not surprisingly, these enzymes are involved in several biological processes such as immune and inflammatory responses, blood pressure regulation and pregnancy maintenance^12,13^. There is also increasing evidence that LNPEP is involved with preterm delivery due to its oxytocinase activity^14^.

Johnson and colleagues identified a quantitative trait locus (QTL) for preeclampsia on chromosome 5q, in a region harboring the aminopeptidases genes^15^ and, subsequently, confirmed the genetic association between *ERAP2* and preeclampsia^16^. The missense genetic variants in *ERAP1*, rs27044 and rs30187, have consistently been reported as associated with ankylosing spondylitis, psoriasis, multiple sclerosis and Crohn’s disease^17^. Lastly, maternal *LNPEP* variants were reported as associated with increased risk of preterm birth^18^. Thus, the present study aimed to evaluate genetic variants in *ERAP1*, *ERAP2* and *LNPEP* for association with the full clinical spectrum of HDP. For the first time, eclampsia and HELLP phenotypes, which are the most severe and rare phenotypes, were tested for these genes.

## Methods

### Population and study design

Our study population was recruited from Maternidade Escola Januário Cicco, a tertiary center for women’s health, located in Natal, Rio Grande do Norte state, Brazil. A total of 1693 women were recruited from 2002 to 2010, as part of a broader study aiming to investigate clinical, epidemiological and genetic aspects of hypertensive disorders of pregnancy. Clinical data as well as blood samples were collected at the time of enrollment. For the current study, we retrospectively selected 1282 women based on their pregnancy outcome: 693 normotensive women (control), 342 preeclampsia (PE), 61 superimposed preeclampsia (PEsuper), 74 eclampsia, and 112 HELLP syndrome cases. All Methods were performed in accordance with the Declaration of Helsink and followed the Brazilian ethical standards of scientific research. The research protocol was reviewed and approved by the Federal University of Rio Grande do Norte (CEP-UFRN 88) and Brazilian National Ethical Committee (CONEP 5059). All research participants or their legal guardian provided informed consent.

### Phenotype definition

The diagnostic criteria followed the recommendations from the American College of Obstetrician and Gynecologists^19^. Preeclampsia was defined as the new onset hypertension (SBP ≥ 140 mmHg or DBP ≥ 90 mmHg) and proteinuria (≥ + 1 on dipstick) after 20 weeks of gestation. Superimposed preeclampsia occurred when the woman had a previous diagnosis of chronic hypertension and developed proteinuria after 20 weeks of gestation. Eclampsia was defined by the presence of seizure, while HELLP syndrome diagnosis was based on Mississippi Class III system (AST > 40 IU/L and LDH > 600 IU/L and platelets < 150,000/μL)^20^. Controls were healthy pregnant women with no history of hypertension. Women with multiple pregnancies, diabetes or other chronic diseases were excluded from study.

### Genetic variants

The variants in *ERAP2* (rs2549796, rs2927609, rs11135484) and *LNPEP* (rs27300, rs38034, rs2303138) were all tag-variants, identified through a pairwise selection strategy with an r^2^ threshold ≥ 0.8 in Haploview 4.2^21^ using the HapMap CEU population genotype data (HapMap Rel 27 phase II + III). Variants rs30187 and rs27044, in *ERAP1*, were chosen based on their effect on protein function^22,23^ as well as their implication in other diseases^17^.

### Genotyping

DNA extraction was carried out as previously described^24^. Samples were genotyped by SNaPshot technique and the capillary electrophoresis performed on ABI PRISM 3100 Avant Genetic Analyzer (Applied Biosystems). Technique standardization was carried out according to Lins and colleagues^25^. GeneMapper software (Applied Biosystems, CA, USA) was used for the genotype calling.

### Population stratification assessment

To avoid confounding by ethnicity we used a panel with 27 ancestry informative markers (AIMs) particularly designed for the Brazilian population^26^. A sub-sample of 756 women randomly selected was used to assess the genetic ancestry of our study population (n = 1282) using principal component analysis in SNPRelate R package^27^. Samples from The 1000 Genomes Project^28^ of European (IBS), African (ASW, MSL, YRI) and American (CLM) origins were used as reference populations.

### Functional validation

Variant effects on mRNA and protein levels were assessed from GTEx (dbGaP Accession phs000424.v8.p2)^29^ and single nucleotide polymorphisms annotator (SNiPA)^30^ databases. Aiming to functionally validate the *ERAP1* genetic finding, we recruited an additional cohort of 65 pregnant women, including 29 normotensive controls and 36 severe preeclampsia cases, that had their Ang II plasma concentration measured by ELISA commercial kit (MyBioSource, San Diego, CA, USA, Cat.Num. MBS453098). Briefly, blood samples were systematically collected between 7 and 9 am in EDTA tubes and immediately centrifuged. The obtained plasma was stored at − 80 °C until assay.

### Statistical analysis

Clinical and demographic data were analyzed through chi-squared and t-test for categorical and quantitative variables, respectively. With regard to the genetic data, allele frequencies were compared by Fisher exact test, whereas genotype and haplotype association tests were performed through logistic regression models including maternal age and parity (primigesta *vs* others) as covariates. Haplotype frequencies were estimated by Expectation–Maximization algorithm with a minor haplotype frequency threshold of 0.03. The *p*-values were corrected for family-wise error rate by permutation procedures (10,000 ×) implemented in PLINK^31^. All analyses were performed by comparing each case phenotype (*i.e.* PE, PEsuper, eclampsia and HELLP) against the normotensive control group.

## Results

### Demographics and clinical characteristics

Table 1 summarizes the main clinical characteristics and demographics for our study population. Women with eclampsia and HELLP were the youngest and oldest, respectively, when compared to the control group, whereas the proportion of primiparas was higher in the preeclampsia and eclampsia groups. Women with HELLP syndrome delivered their babies earlier in pregnancy (mean gestational age = 34.3 weeks), followed by eclampsia (mean gestational age = 36.2 weeks) and preeclampsia (37.2 weeks) groups. Overall, the frequency of family members affected by chronic hypertension was higher in the case groups when compared to control group, suggesting shared genetic components between essential hypertension and hypertensive disorders of pregnancy (Table 1). Of note, the prevalence of chronic hypertension and eclampsia in family members was much higher in superimposed preeclampsia (PEsuper) and eclampsia groups, respectively.

**Table 1:** Demographics and clinical characteristics.

Characteristics	Control	PE	PEsuper	Eclampsia	HELLP
Sample size, n	693	342	61	74	112
Maternal age, y, mean (± SD)	24.4 (± 6.2)	25.2 (± 6.8)	31.1 (± 6.9)^a^	20.5 (± 6.1)^a^	27.0 (± 6.7)^a^
SBP, mmHg, mean (± SD)	117 (± 12)	156 (± 19)^a^	166 (± 28)^a^	159 (± 21)^a^	154 (± 21)^a^
DBP, mmHg, mean (± SD)	75 (± 9)	105 (± 13)^a^	108 (± 14)^a^	108 (± 16)^a^	101 (± 13)^a^
**Proteinuria, n (%)**					
Negative	693 (100)	0 (0.0)	0 (0.0)	2 (2.7)	7 (6.3)
1 +	–	104 (30.4)	28 (45.9)	11 (14.9)	13 (11.6)
≥ 2 +	–	238 (69.6)	16 (26.2)	43 (58.1)	85 (75.8)
Missing	–	0 (0.0)	17 (27.9)	18 (24.3)	7 (6.3)
Primigestas, n (%)	300 (43.3)	180 (52.8)^a^	14 (23.3) ^a^	54 (73.0)^a^	46 (41.4)
Gestational age at delivery, w, mean (± SD)	38.8 (± 3.1)	37.2 (± 3.0)^a^	35.8 (± 3.5)^a^	36.2 (± 3.6)^a^	34.3 (± 4.0)^a^
Number of antenatal care visits, mean (± SD)	5.9 (± 2.8)	6.2 (± 2.3)	6.1 (± 2.9)	4.5 (± 2.5)^a^	5.3 (± 2.3)^a^
**Family history of**					
Chronic hypertension^b^	238 (35.5)	178 (56.9)^a^	46 (80.7)^a^	32 (46.4)	55 (49.6)^a^
Preeclampsia^c^	22 (3.8)	41 (15.9)^a^	5 (10.9)^a^	12 (21.0)^a^	8 (8.3)^a^
Eclampsia^c^	7 (1.2)	11 (4.4)^a^	3 (6.5)^a^	5 (8.8)^a^	2 (2.1)

*SBP/DBP* systolic blood pressure/diastolic blood pressure.

*PEsuper*: chronic hypertension with superimposed preeclampsia.

^a^*p* < 0.05 for comparison with the control group.

^b^If at least one first degree relative has the disease.

^c^If the mother had had the disease.

### Genetic analysis

All genetic variants achieved standard quality control thresholds (*i.e.* genotyping error rates < 0.05, minor allele frequency > 0.01, and *p*-value > 0.05 for Hardy–Weinberg equilibrium test). In addition, there was no evidence of population stratification, since cases and controls were equally distributed across the reference ethnical groups (Supplementary Fig. 1).

**Figure 1:**
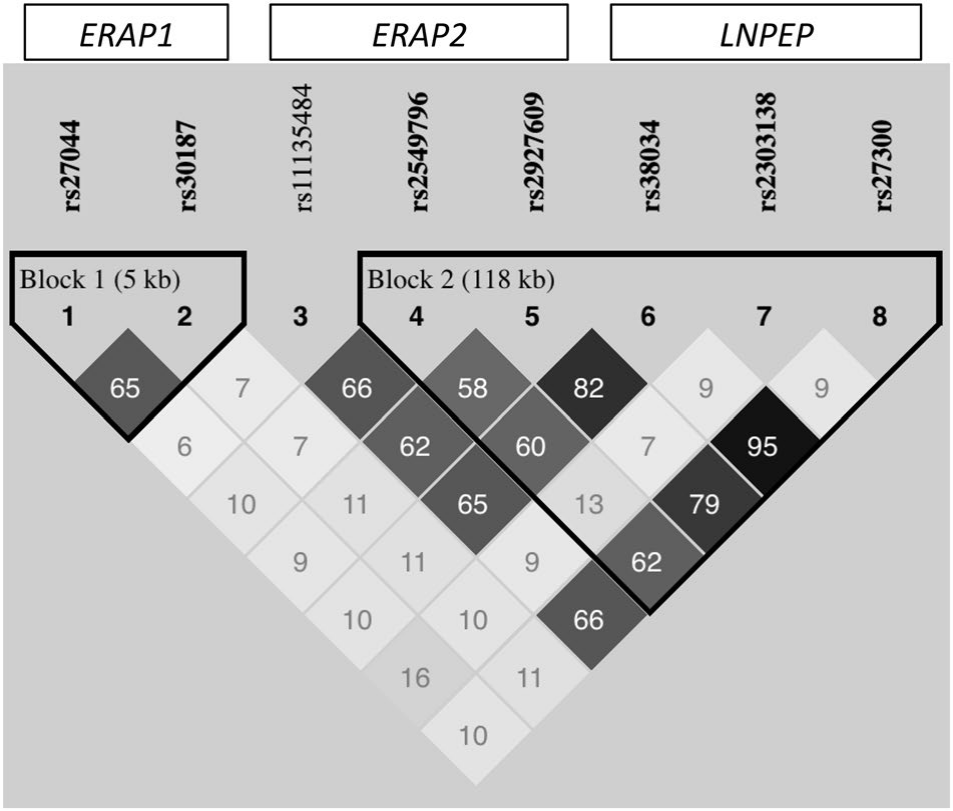
Linkage disequilibrium (LD) pattern among the studied markers. In the LD plot, the inside-square number represents the correlation coefficient value (r^2^). The genomic organization and LD pattern suggest *ERAP2* and *LNPEP* markers as belonging to the same haploblock.

Figure 1 shows the linkage disequilibrium (LD) pattern across the genomic region encompassing the studied variants. *ERAP1* variants were moderately correlated (r^2^ = 0.65) while *ERAP2* and *LNPEP* variants seemed to belong to the same haploblock.

In order to analyze the combined effect of variants on disease risk, we performed haplotype-based tests (Table 2). As result, *ERAP2* haplotype rs2549796–rs2927609–rs11135484 was associated with preeclampsia (corrected *p* = 0.0109). There was no haplotype associated with the remaining case groups (corrected *p* > 0.05).

**Table 2:** Haplotype tests of association showing *ERAP2 C–C–G* haplotype associated with preeclampsia.

Haplotypes	Haplotype frequency				
	Control	PE	PEsuper	Eclampsia	HELLP
***ERAP1***					
T–G	0.354	0.358	0.328	0.341	0.323
T–C	0.105	0.097	0.121	0.051	0.074
C–C	0.541	0.545	0.552	0.609	0.602
***ERAP2***					
C–T–A	0.362	0.361	0.356	0.301	0.352
C–C–A	0.064	0.066	0.060	0.065	0.079
C–C–G	0.045	**0.076**^**a**^	0.050	0.045	0.065
T–C–G	0.488	0.482	0.515	0.523	0.452
***LNPEP***					
T–C–A	0.123	0.130	0.105	0.132	0.135
C–T–G	0.398	0.376	0.375	0.345	0.397
T–C–G	0.479	0.494	0.520	0.521	0.468

*ERAP1*: rs30187–rs27044.

*ERAP2*: rs2549796–rs2927609–rs11135484.

*LNPEP*: rs27300–rs38034–rs2303138.

^a^PE versus Control (Uncorrected *p* = 0.0013; *p*-value corrected for family-wise error rates by running 10,000 permutations: *p* = 0.0109).

There was no difference regarding allele frequencies between control and case groups (Supplementary Table 1), although the genotype distribution for *ERAP1* variants in eclampsia group seemed to differ, when compared to controls (Table 3). The frequency of genotype C/C (rs30187) was notably higher in eclampsia group (40.9%), what would be consistent with a recessive genetic model.

**Table 3:** Genotype distribution for *ERAP1* variants across phenotypic groups.

SNP	Genotypes	Genotype distribution, n (%)				
		Control	PE	PEsuper	Eclampsia^a^	HELLP
rs30187	T/T	139 (20.5)	68 (20.5)	12 (19.7)	16 (22.5)	16 (14.6)
	C/T	343 (50.7)	167 (50.5)	32 (52.5)	26 (36.6)	56 (50.9)
	C/C	195 (28.8)	96 (29.0)	17 (27.8)	29 (40.9)	38 (34.6)
rs27044	G/G	86 (12.7)	48 (14.5)	9 (15.5)	14 (19.2)	12 (10.9)
	G/C	307 (45.4)	141 (42.6)	20 (34.5)	23 (31.5)	48 (43.6)
	C/C	283 (41.9)	142 (42.9)	29 (50.0)	36 (49.3)	50 (45.5)

^a^Chi-squared test of genotypic association for Control vs Eclampsia: *p* = 0.055 for rs30187 and *p* = 0.079 for rs27044.

Of note, rs30187 C allele codes for Arg528 (instead of Lys528), resulting in an enzyme type characterized by lower peptidase activity against Ang II^32^. Therefore, we defined a recessive genetic model with C/C as the risk genotype for eclampsia. The model was implemented through logistic regression with genotype (T/T + T/C vs C/C) as the main explanatory variable and maternal age, and parity as covariates. As result, women homozygotes for rs30187 C/C risk genotype were more likely to develop eclampsia (OR = 1.85, *p* = 0.019) (Table 4).

**Table 4:** Genetic effect of rs30187 on eclampsia risk under recessive genetic model.

Genotypic group	Control^a^	Eclampsia^a^	Eclampsia risk^b^	
	n (%)	n (%)	OR (95% CI)	*p*-value
T/T + C/T	482 (71.2)	40 (58.0)	1.0	0.019
C/C	195 (28.8)	29 (42.0)	1.85 (1.11–3.11)	

^a^Chisquared test of association for genotype distribution between Control and Eclampsia (*p* = 0.022).

^b^Odds ratio (OR) and confidence interval (CI) estimated by logistic regression model adjusted for maternal age and parity.

### Functional validation

**Figure 2:**
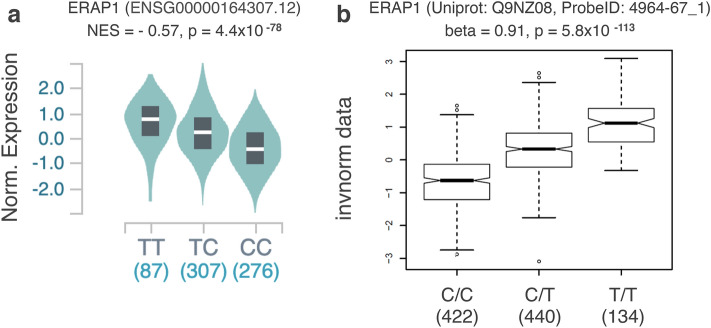
Effect of *ERAP1* rs30187 variant on mRNA (Data source: GTEx) and protein levels (Data source: SNiPA) in blood. *ERAP1* rs30187 CC genotype is associated with the lowest expression level. Each T allele additivelly increases *ERAP1* mRNA in whole blood cells (**a**) and Erap1 protein concentration in plasma (**b**). NES: Normalized effect size; Norm. Expression: normalized expression; invnorm data: inverse-normal scaled data, (**b**) was kindly provided by Karsten Suhre from SNiPA team.

According to GTEx and SNiPa data, rs30187 has a significant effect on both ERAP1 mRNA and protein levels in blood with C/C genotype associated with the lowest expression levels (Fig. 2).

**Figure 3:**
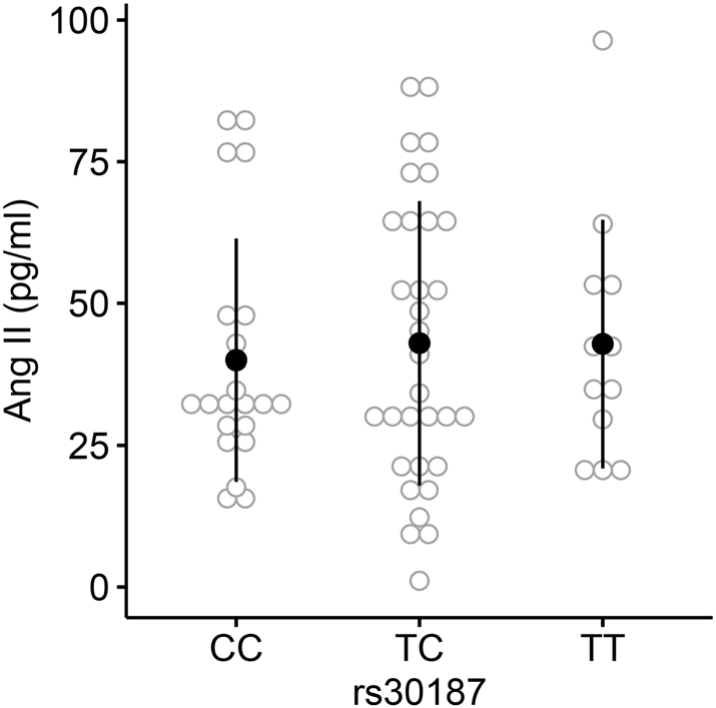
Plasma Ang II levels across rs30187 genotypic groups. Analysis of plasma Ang II concentration in pregnant women (n = 65). No difference was detected among genotypic groups (ANOVA, *p* = 0.895).

Given the qualitative and quantitative effect of rs30187 on ERAP1 expression, we hypothesized that women homozygotes for C/C genotype have higher circulating levels of Ang II. In order to test that, plasma Ang II concentrations were determined in an additional cohort of women with severe preeclampsia (n = 36) and normotensive pregnant controls (n = 29). We rejected this hypothesis (*p* = 0.895) since no difference was detected between genotypic groups (Fig. 3). The intra-group analysis (*i.e.* cases-only and controls-only) did not detect any difference in Ang II levels across genotypic groups as well (data not shown).

## Discussion

Endoplasmic reticulum aminopeptidases (ERAPs), as well as leucyl/cystinyl aminopeptidase (LNPEP), play roles in antigen processing, inflammatory response, blood pressure regulation and angiogenesis, all processes potentially implicated in preeclampsia pathophysiology. The present study confirmed a genetic association between *ERAP2* and preeclampsia, and, for the first time, reported an association between *ERAP1* and eclampsia. These findings may help to disentangle the intricate association between the correlated phenotypes preeclampsia/eclampsia and the functionally and physically connected genes *ERAP1*/*ERAP2*. In addition, our results are consistent with the hypothesis of distinct genetic bases for preeclampsia and eclampsia, reinforcing the importance to separate the two phenotypes when designing genetic association studies.

Johnson et al., tested ERAP1, ERAP2 and LNPEP for associations with preeclampsia in Australian and Norwegian populations, and identified *ERAP2* variants (rs2549782, rs2548538, rs2287988 and rs17408150) associated with preeclampsia^16^. In the same study, rs27044 and rs30187 (*ERAP1*) were not associated with disease although borderline association with preeclampsia was found for markers rs3734016 and rs34750, both within *ERAP1* gene. It is important to highlight that the Australian cohort contained both preeclampsia and eclampsia cases, but they were analyzed as a unique group^16^, while our study treated the two phenotypes as different entities. A recent study with 148 preeclamptic women and 133 controls from Iran investigated four variants in *ERAP1* (including rs30187) and three variants in *ERAP2*. None of the variants were associated with disease, but a haplotype encompassing the seven variants was associated with preeclampsia^33^. In another Iranian independent study, *ERAP2* variants (rs2549782 and rs17408150) were also associated with preeclampsia^34^. In our study, the *ERAP2* variants rs2549796, rs2927609 and rs11135484 were not associated with preeclampsia in a single marker analysis but the haplotype C–C–G was overrepresented in the preeclampsia group (Table 2). Interestingly, the fetal minor allele for variant rs2549782 (*ERAP2*) was associated with preeclampsia in African American population^35^. Besides the genetic association findings, Founds et al. showed *ERAP2* was differentially expressed in the first trimester placentas of women who later developed preeclampsia^36^.

The *ERAP1* rs30187 C allele codes an enzyme with Arg528 that causes a reduction on peptidase activity for angiotensin II degradation by approximately 60%, when compared to the enzyme with Lys528, coded by the T allele^22,32^. We failed to confirm the hypothesis that women carrying two copies of the C allele have increased levels of Ang II in their blood, which in turn could cause blood pressure elevation and seizure. However, we cannot rule out a potential effect of rs30187 on local RAS (*e.g.* brain and kidney). While Arg528 variant is associated with hypertensive disease^37^, the Lys528 variant is strongly associated with susceptibility to ankylosing spondylitis^38^ and other autoimmune diseases^17^. Since both Arg528 and Lys528 alleles are associated with bad outcomes, it is likely that *ERAP* genes would be subject to balancing selection, a process where heterozygous individuals are more adaptive than either of the two types of homozygous^39^. In addition, these genes play key role in the maintenance of immunotolerance to self-peptides as well as protecting against infectious agents, such as HIV^40^.

The small sample size for some of our case groups represents an important limitation for the present study, even though we should consider that eclampsia and HELLP are extremely rare phenotypes. On the other hand, the marker associated with eclampsia (rs30187) has been well characterized as affecting the protein function, what strengthens the biological plausibility for the genetic association reported here. Furthermore, we accounted for important confounders such as age, parity and ethnicity.

The mechanism by which endoplasmic reticulum aminopeptidases (ERAPs) influence the risk of preeclampsia/eclampsia remains to be elucidated. In addition to Ang II degradation and peptide trimming for antigen presentation via MHC-1, ERAPs also play role in inflammatory response by shedding cytokine receptors (*e.g.* IL-6R, IL-1R2 and TNFR)^41–43^. Lastly, *ERAP1* plays crucial role in VEGF-stimulated proliferation and migration of endothelial cells, as well as angiogenesis, via the binding and modification of PDk1^44^. All the above-mentioned mechanisms are potentially involved in the causal pathway of preeclampsia/eclampsia.

## Conclusions

In conclusion, we identified genetic variants in *ERAP1* and *ERAP2* associated with eclampsia and preeclampsia, respectively. Sequencing and functional studies are needed in order to elucidate the mechanisms underlying these genetic associations.

## Supplementary Information

Supplementary Information 1.

## Data Availability

The genetic data used in the present study is available from the corresponding author on reasonable request.
