# Supplementary material for: Genetic association of ERAP1 and ERAP2 with eclampsia and preeclampsia in northeastern Brazilian women
Source: Sci Rep. 2021 Mar 24;11:6764. doi: 10.1038/s41598-021-86240-z (PMC7990956; doi:10.1038/s41598-021-86240-z)
Supplement: Supplementary file 1 — Supplementary Information 1. [file 41598_2021_86240_MOESM1_ESM.pdf]

Supplementary Information

Title: Genetic association of *ERAP1* and *ERAP2* with eclampsia and preeclampsia in northeastern Brazilian women

LEONARDO CAPISTRANO FERREIRA * (ferreiralc@cb.ufrn.br)
Department of Biochemistry, Federal University of Rio Grande do Norte, Natal, Brazil; Institute of Tropical Medicine of Rio Grande do Norte, Federal University of Rio Grande do Norte, Natal, Brazil.

CARLOS EDUARDO MAIA GOMES (c_maia@yahoo.com)
Department of Biophysics and Pharmacology, Federal University of Rio Grande do Norte, Natal, Brazil.

PRIYA DUGGAL (pduggal@jhu.edu)
Bloomberg School of Public Health, Johns Hopkins University, Baltimore, USA.

INGRID DE PAULA HOLANDA
Department of Biochemistry, Federal University of Rio Grande do Norte, Natal, Brazil.

AMANDA SAMARA DE LIMA
Maternidade Escola Januario Cicco, Federal University of Rio Grande do Norte.

PAULO RICARDO PORFÍRIO DO NASCIMENTO
Institute of Tropical Medicine of Rio Grande do Norte, Federal University of Rio Grande do Norte, Natal, Brazil.

SELMA MARIA BEZERRA JERONIMO (smbj@cb.ufrn.br)
Department of Biochemistry, Federal University of Rio Grande do Norte, Natal, Brazil; Institute of Tropical Medicine of Rio Grande do Norte, Federal University of Rio Grande do Norte, Natal, Brazil; Institute of Science and Technology of Tropical Diseases (INCT-DT), Brazil.

*** Corresponding author:**
Leonardo Capistrano Ferreira
Department of Biochemistry, Center of Biosciences, Federal University of Rio Grande do Norte. Av. Sen. Salgado Filho, S/N, Campus Universitário - Lagoa Nova - Natal, RN, 59078-900, Brazil.
E-mail: ferreiralc@cb.ufrn.br

Supplementary Information

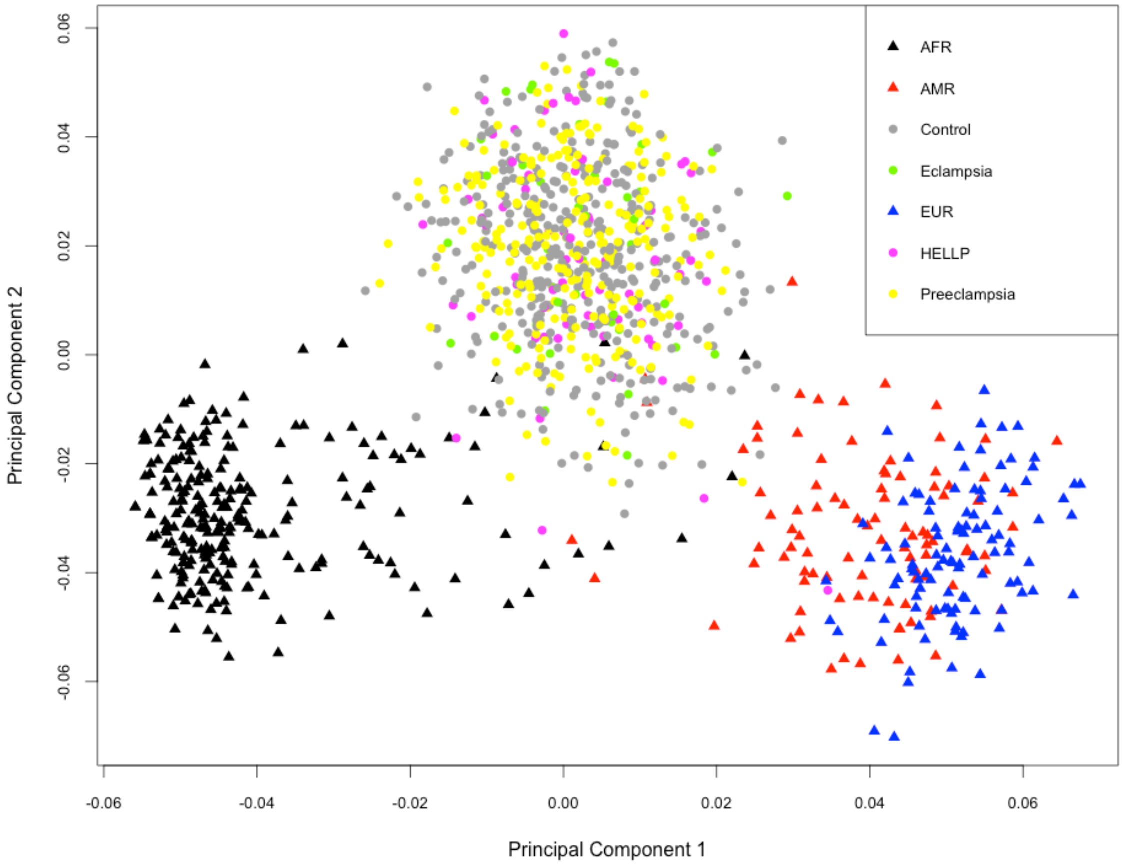

Supplementary Figure 1. Scatter plot of the two principal components estimated from genotype information of 27 Ancestry Informative Markers (AIMs). Our study samples (n=756) belong to the highly admixed Brazilian population that clustered together in an intermediate region relative to the African (AFR), European (EUR) and Amerindian (AMR) reference populations from The 1000 Genomes Project. Importantly, control and cases women were equally distributed suggesting absence of population stratification.

Supplementary Information

Supplementary Table 1. Allele frequency comparisons across phenotypic groups.

Gene	SNP	Minor allele	Minor allele frequency				
			Control	PE	PEsuper	Eclampsia	HELLP
LNPEP	rs27300	C	0.402	0.382	0.375	0.370	0.414
	rs38034	T	0.396	0.371	0.375	0.340	0.389
	rs2303138	A	0.122	0.130	0.105	0.132	0.128
ERAP1	rs30187	T	0.459	0.458	0.459	0.391	0.400
	rs27044	G	0.354	0.358	0.328	0.340	0.327
ERAP2	rs2549796	C	0.485	0.506	0.467	0.406	0.509
	rs2927609	T	0.380	0.364	0.356	0.326	0.385
	rs11135484	A	0.447	0.443	0.440	0.425	0.467

No difference was detected ($p>0.05$) when each case group was compared against control group.
